# Probing Resistance Mutations in Retroviral Integrases by Direct Measurement of Dolutegravir Fluorescence

**DOI:** 10.1038/s41598-017-14564-w

**Published:** 2017-10-25

**Authors:** Eloïse Thierry, Samuel Lebourgeois, Françoise Simon, Olivier Delelis, Eric Deprez

**Affiliations:** Laboratory of Biology and Applied Pharmacology (LBPA), CNRS UMR8113, IDA FR3242, ENS Paris-Saclay, Université Paris-Saclay, F-94235 Cachan, France

## Abstract

FDA-approved integrase strand transfer inhibitors (raltegravir, elvitegravir and dolutegravir) efficiently inhibit HIV-1 replication. Here, we present fluorescence properties of these inhibitors. Dolutegravir displays an excitation mode particularly dependent on Mg^2+^ chelation, allowing to directly probe its Mg^2+^-dependent binding to the prototype foamy virus (PFV) integrase. Dolutegravir-binding studied by both its fluorescence anisotropy and subsequent emission enhancement, strictly requires a preformed integrase/DNA complex, the ten terminal base pairs from the 3′-end of the DNA reactive strand being crucial to optimize dolutegravir-binding in the context of the ternary complex. From the protein side, mutation of any catalytic residue fully abolishes dolutegravir-binding. We also compared dolutegravir-binding to PFV F190Y, G187R and S217K mutants, corresponding to HIV-1 F121Y, G118R and G140S/Q148K mutations that confer low-to-high resistance levels against raltegravir/dolutegravir. The dolutegravir-binding properties derived from fluorescence-based binding assays and drug susceptibilities in terms of catalytic activity, are well correlated. Indeed, dolutegravir-binding to wild-type and F190Y integrases are comparable while strongly compromised with G187R and S217K. Accordingly, the two latter mutants are highly resistant to dolutegravir while F190Y shows only moderate or no resistance. Intrinsic fluorescence properties of dolutegravir are thus particularly suitable for a thorough characterization of both DNA-binding properties of integrase and resistance mutations.

## Introduction

Integration of the HIV-1 genome into the host genome is a crucial event in the retrovirus life cycle and corresponds to a two-step reaction catalysed by integrase (IN)^1–3^. The first step corresponds to the 3′-processing reaction (3′P) that involves cleavage of the 3′- terminal dinucleotide at each viral DNA end. The hydroxyl groups of newly recessed 3′-ends are used in the second step, named strand transfer (ST) for the covalent joining of viral and target DNAs, resulting in full-site integration.

IN strand transfer inhibitors (INSTIs)^3–6^, together with allosteric inhibitors of IN^7–13^, efficiently inhibit viral replication. Allosteric inhibitors correspond to noncatalytic site inhibitors of IN and may interfere with distinct steps than integration, whereas INSTIs target the active site of IN and consistently inhibit the overall integration process by specifically blocking the ST reaction. To date, only INSTI compounds have been developed for use in patients. Among them, Raltegravir (RAL) and elvitegravir (EVG) (first generation of IN inhibitors) as well as dolutegravir (DTG) (second generation) are three potent INSTIs approved by the US FDA^14–18^. Regarding RAL-resistance mutations, three resistance pathways have been identified, involving primary mutations at positions Y143, Q148 and N155 in the HIV-1 IN (IN^HIV^) sequence^19–21^. If EVG displays extensive cross-resistance with RAL, the second-generation DTG compound which is intrinsically more potent against IN, leads to efficient inhibition of N155 and Y143 pathways, albeit some resistance to DTG may be associated with the Q148 pathway^22–24^. To date, there is no specific resistance pathway identified in DTG-treated patients. Recently, we have identified and characterized two novel single mutations, G118R and F121Y, originally described in patients failing RAL-containing regimens, that also confer resistance against DTG, however, to different extents (G118R»F121Y)^24^.

Although there is no available 3D X-ray structure of the full-length IN^HIV^ (free or DNA-bound) for solubility reasons, several X-ray structures of the more soluble full-length prototype foamy virus (PFV) IN (IN^PVF^)^25^ are now available in complex with a pair of viral DNA ends^26^, also bound to target DNA^27^, and several structures exist in the presence of INSTI such as RAL and EVG^26,28^ as well as with second-generation DTG and MK2048 inhibitors^28,29^. The structure of a PFV intasome/nucleosome complex at 7.8 Å-resolution obtained by cryo-electron microscopy is also available^30^. IN^PFV^ structure is a valuable model for investigating many properties of IN^HIV^, including catalytic mechanism of integration, IN-DNA interactions (with both donor and target DNAs) as well as interactions with INSTIs. In the latter case, IN^PVF^-inhibitor complexes may provide platforms for structure-based design of new inhibitors with reduced susceptibilities to resistance mutations^31^.

Here, we present fluorescence properties of RAL, EVG and DTG. Among them, DTG particularly displays interesting fluorescence emission characteristics. Although DTG alone in aqueous solution was poorly fluorescent *per se*, a significant fluorescence enhancement was specifically observed upon complexation with Mg^2+^. This is relevant in the context of IN since (i) Mg^2+^ as a metallic divalent cation represents the physiological cofactor for IN activity and a pair of metal cations is visible in the active site of published structures, coordinated to the catalytic triad motif (DD35E) between the side chains of D128/D185 and D128/E221, respectively^26,28^, (ii) in addition to drug/amino-acid interactions, three co-planar atoms (mainly oxygen) of INSTIs are coordinated to both metal ions in the active site, the central oxygen being coordinated to both ions^26,28,29^ and define the metal-coordinating pharmacophore which together with the halogenated phenyl ring are key structural features of this anti-IN compound family.

The Mg^2+^-dependent IN-DTG interaction was then studied by two fluorescence-based strategies, *i.e*. fluorescence emission enhancement and fluorescence anisotropy of DTG. Both approaches were compatible with DTG concentrations of 0.3–0.6 μM in terms of detection sensitivity. This typical concentration range was suitable for studying IN^PFV^ but critical in the case of IN^HIV^ for solubility reasons as underlined above^25,32,33^. Consequently, a similar analysis was unsuccessful with IN^HIV^. Taking into account that the comparison of full-length IN^PFV^ and truncated IN^HIV^ structures highlights common structural features^26,34,35^ and that both proteins share common catalytic mechanisms and behave similarly toward anti-IN drugs, including INSTI^25,32,36^, the predictive value of IN^PFV^ is supposed to be high. Our findings define a new approach to study the binding properties of DTG to IN and reach deeper insight into the mechanisms of drug resistance mutations such as G187R, F190Y and S217K (equivalent to G118R, F121Y and G140S/Q148K mutations, respectively, in IN^HIV^).

## Results and Discussion

### DTG fluorescence emission is strongly dependent on Mg^2+^

**Figure 1:**
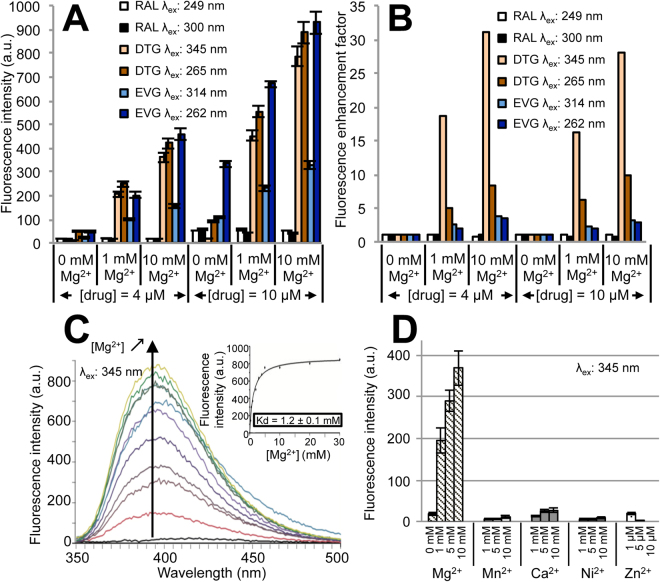
Fluorescence properties of INSTIs in solution and influence of divalent cations. Fluorescence emission intensities (**A**) or fluorescence enhancement factors (**B**) of RAL (λ_max,em_ = 363 nm), EVG (λ_max,em_ = 354 nm) and DTG (λ_max,em_ = 395 nm) as a function of the excitation wavelength, drug and Mg^2+^ concentrations. EVG and DTG were studied in buffer A whereas RAL was studied in buffer A-10% EtOH (v/v). Corresponding excitation and emission spectra are shown in Supplementary Fig. S1. Excitation and emission slits: Δλ_ex_ = Δλ_em_ = 5 nm (PMT = 750 V). The fluorescence enhancement factor corresponds to the drug fluorescence emission intensity for a given Mg^2+^ concentration normalized by the intensity in the absence of Mg^2+^. (**C**) Fluorescence emission spectra of DTG (1 μM) in the presence of increasing Mg^2+^ concentrations (λ_ex_ = 345 nm; Δλ_ex_ = Δλ_em_ = 5 nm; PMT = 960 V). The emission intensity was reported as a function of [Mg^2+^] (inset). The graph shows representative data of three independent experiments. (**D**) Fluorescence intensity enhancement of DTG is specific of Mg^2+^ chelation. Emission intensity of DTG (0.3 μM) at 395 nm upon addition of various divalent cations (λ_ex_ = 345 nm; Δλ_ex_ = Δλ_em_ = 5 nm; PMT = 980 V). The bar graphs in panels A and D show mean ± SD values from three independent experiments.

We first investigated the fluorescence properties of three INSTIs (RAL, DTG, EVG) when free in solution and the influence of Mg^2+^ on drug fluorescence emission. In aqueous solution (Tris-buffer A), all drugs exhibited two excitation modes, at 249/320 nm for RAL, 265/345 nm for DTG and 262/314 nm for EVG, corresponding to S_0_→S_2_ and S_0_→S_1_ transitions, respectively (Supplementary Fig. S1). The wavelengths of maximum emission intensity were 405, 395 and 354 nm, for RAL, DTG and EVG, respectively. Significant blue-shifts of one excitation mode of RAL (320→300 nm) and its emission wavelength (405→363 nm) with a net increase in the fluorescence intensity was observed in the presence of ethanol only (buffer A-EtOH 10% (v/v)) (Supplementary Fig. S1). However, RAL was characterized by a very low fluorescence emission, regardless of Mg^2+^ concentration (Fig. 1A).

By contrast, EVG and DTG were intrinsically more fluorescent in buffer A and excitation at 262/265 nm led to higher fluorescence than excitation at 314/345 nm (Fig. 1A). Furthermore, both drugs display a Mg^2+^ dependence of fluorescence intensity, however with some differences. A slight increase of EVG fluorescence intensity was observed upon addition of Mg^2+^ (a 3-fold increase for the two excitation modes from 0 to 10 mM Mg^2+^; Fig. 1B). DTG fluorescence emission was more Mg^2+^-dependent than EVG with, interestingly, the second excitation mode (345 nm) leading to a stronger Mg^2+^ dependence of emission intensity than the first one (265 nm) (a 27–30-fold and a 8–10-fold increase at 345 and 265 nm, respectively, from 0 to 10 mM Mg^2+^; Fig. 1B). The K_d_ value relative to the DTG-Mg^2+^ interaction was about 1.2 mM (calculated from the complete hyperbolic titration curve: enhancement of DTG emission as a function of [Mg^2+^]; Fig. 1C). Note that, among the different divalent cations tested (Mg^2+^, Mn^2+^, Ca^2+^, Ni^2+^, Zn^2+^), only Mg^2+^ strongly enhanced DTG fluorescence emission (Fig. 1D). This Mg^2+^-dependent enhancement is related to an increase of the fluorescence quantum yield since no Mg^2+^-dependent change in the absorption spectrum was observed (Supplementary Fig. S2A). Moreover, the increase of medium viscosity mimics the Mg^2+^ effect in terms of fluorescence enhancement (Supplementary Fig. S2B): only EVG and DTG displayed a glycerol-dependent fluorescence enhancement, showing that molecular geometry (planarity) and internal dynamics accounts for the quantum yield increase, although the particularly high fluorescence enhancement of DTG in the 345nm-excitation mode appears to be specific of Mg^2+^ chelation.

This large Mg^2+^-dependent enhancement of DTG fluorescence intensity upon excitation at 345 nm was used in the following sections to directly probe drug binding to IN since (i) Mg^2+^ is the physiologically relevant catalytic cofactor coordinated in the protein active site and (ii) INSTI binding to the active site is dependent on Mg^2+^ according to available X-ray structures^26,28,29^.

### DTG binds to the pre-established binary IN-Viral DNA complex but not to IN alone

Taking into account that the K_d_ values characterizing DTG-Mg^2+^ (Fig. 1C) and IN-Mg^2+^ interactions (as estimated by monitoring the 3′P activity of IN as a function of [Mg^2+^]; Supplementary Fig. S3) are similar (in the 1–2 mM range), we reasoned that 1 mM Mg^2+^ (around the K_d_ values) corresponds to an optimal condition to measure the highest fluorescence enhancement upon DTG binding to IN. Indeed, only the binding of uncomplexed DTG to IN-Mg^2+^ is expected to increase the fluorescence signal. The binding of DTG-Mg^2+^ or uncomplexed DTG to uncomplexed IN, or the solvent→IN transition of DTG, when both DTG and IN are initially complexed to Mg^2+^, is not expected to change the net balance of the fluorescence signal. The latter situation precludes the use of saturating concentrations of Mg^2+^ (*e.g*. 10 mM) for measuring DTG binding to DNA-free or DNA-bound IN by fluorescence intensity.

**Figure 2:**
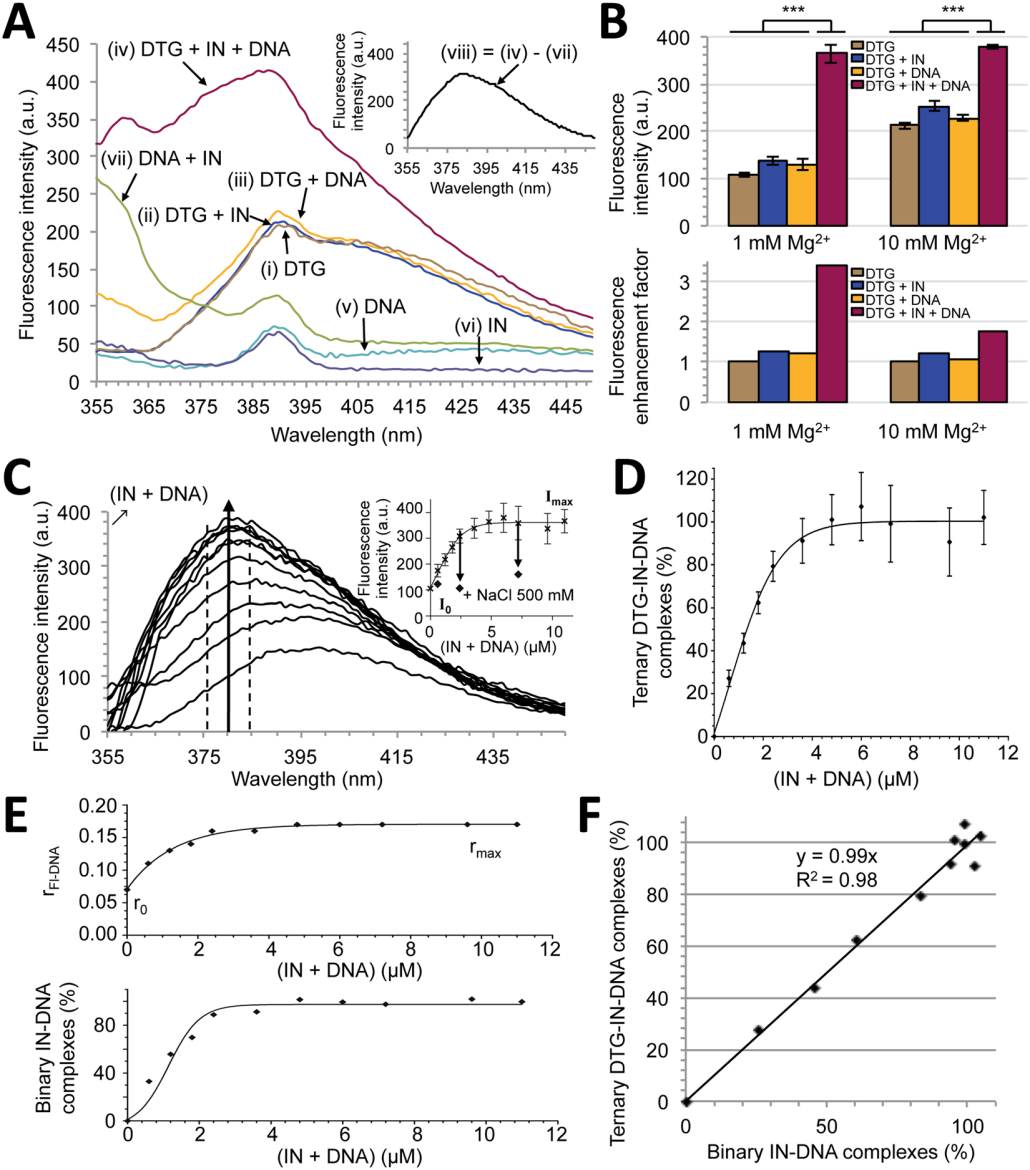
Fluorescence properties of DTG in the context of the binary INPFV-viral DNA complex. (**A**) Fluorescence emission spectra of DTG (0.3 μM) alone (i), in the presence of 1.2 μM IN (ii), 1.2 μM DNA (PFV^CAAT^) (iii) or in the presence of both IN and DNA ([IN] = [DNA] = 1.2 μM) (iv). All experiments were performed in the presence of 1 mM Mg^2+^ (see Supplementary Fig. S4 for [Mg^2+^] = 10 mM). λ_ex_ = 345 nm and PMT = 980 V (Δλ_ex_ = Δλ_em_ = 5 nm). DNA alone (v) or IN alone (vi) displays water Raman scattering peak (390 nm) whereas the stoichiometric IN:DNA mixture (vii) also displays Rayleigh scattering (on the blue side of the spectrum), probably due to the presence of aggregated species. Contributions of Raman and Rayleigh scattering were eliminated in the inset spectrum: (viii) = (iv)–(vii) where (vii) represents the proper spectrum of Mg^2+^-DTG in the context of the binary IN-DNA complex. (**B**) DTG emission intensities (integrated in the 375–385 nm spectral region) under different conditions (top) and corresponding fluorescence enhancement factors (fluorescence intensity normalized by the intensity of DTG alone) (bottom) are reported for two Mg^2+^ concentrations. [DTG] = 0.3 μM; [IN] = [DNA] = 3.6 μM. The bar graph shows mean ± SD values from 3 independent experiments, ***p < 0.001. (**C**) Emission enhancement and spectral shift of DTG (0.3 μM) upon addition of increasing stoichiometric concentrations of (IN + DNA). [Mg^2+^] = 1 mM. Inset: fluorescence intensity (375–385 nm) as a function of (IN + DNA). The arrows indicate the intensity level after addition of 500 mM NaCl. Emission spectra are representative of 3 independent experiments for each condition of (IN + DNA). The graph (inset) shows mean ± SD values from these 3 independent experiments. (**D**) Percentage of ternary DTG-IN-DNA complexes (or % of bound DTG) as a function of (IN + DNA). The % values are derived from the inset figure in panel C: (I–I_0_)/(I_max_–I_0_)x100 where I_0_ and I_max_ correspond to intensities of DTG alone (only Mg^2+^) and in the presence of an excess concentration of (IN + DNA) ( = plateau value in panel C, inset), respectively. (**E**) Measurement of the binary IN-DNA complex formation. The formation of binary complexes was performed by monitoring the fluorescence anisotropy (r) of DNA (Fl-PFV^CAAT^) under similar experimental conditions of the DTG-binding assay (panels C-D). Top, varying stoichiometric concentrations of IN and DNA were mixed and the r parameter was monitored. A constant concentration of Fl-DNA (0.3 μM) was maintained throughout the titration. The graph shows representative data of four independent experiments. Bottom, corresponding percentages of binary complexes as a function of (IN + DNA): % = (r–r_0_)/(r_max_–r_0_)x100 where r_0_ and r_max_ correspond to anisotropy values of free DNA and IN-bound DNA, respectively. (**F**) Linear relationship between the percentages of ternary and binary complexes.

Emission spectra of DTG, alone (i) or in the presence of IN (ii) or PFV^CAAT^ ( = double-stranded 21-mer DNA mimicking the PFV U5 LTR end) (iii) or IN + PFV^CAAT^ (iv), were performed in the presence of 1 mM Mg^2+^ using excitation at 345 nm (Fig. 2A). Only the simultaneous presence in the sample of IN and DNA together with DTG (condition iv) led to a significant increase in the fluorescence emission signal. The three other conditions (i→iii) led to superimposable spectra. To note, the presence of IN/DNA complexes accounts for light scattering on the blue side of the spectrum. The resulting spectrum of DTG bound to the IN-PFV^CAAT^ binary complex is shown in Fig. 2A (inset) after correction of Rayleigh and Raman scattering. The maximum emission wavelength of DTG in the protein context was 381 nm, a significantly blue-shifted maximum compared to DTG in solution (395 nm; see above). As expected, DTG fluorescence enhancement was mainly observed in 1 mM Mg^2+^ (≈3.4-fold) whereas a slight fluorescence enhancement was observed in 10 mM Mg^2+^ after addition of IN + PFV^CAAT^ (≈1.8-fold) (Fig. 2B). DTG fluorescence emission was next monitored in the presence of 1 mM Mg^2+^ and increasing stoichiometric concentrations of (IN + PFV^CAAT^) (Fig. 2C). The fluorescence emission intensity continuously increased and displayed a hyperbolic saturation curve (Fig. 2C, inset) easily converted into a fractional saturation curve (Fig. 2D) confirming that DTG primarily binds to the binary IN-viral DNA complex. DTG binding to the binary complex is reversible by increasing ionic strength (Fig. 2C, inset) which is known to disrupt IN-DNA interactions^25^.

**Figure 3:**
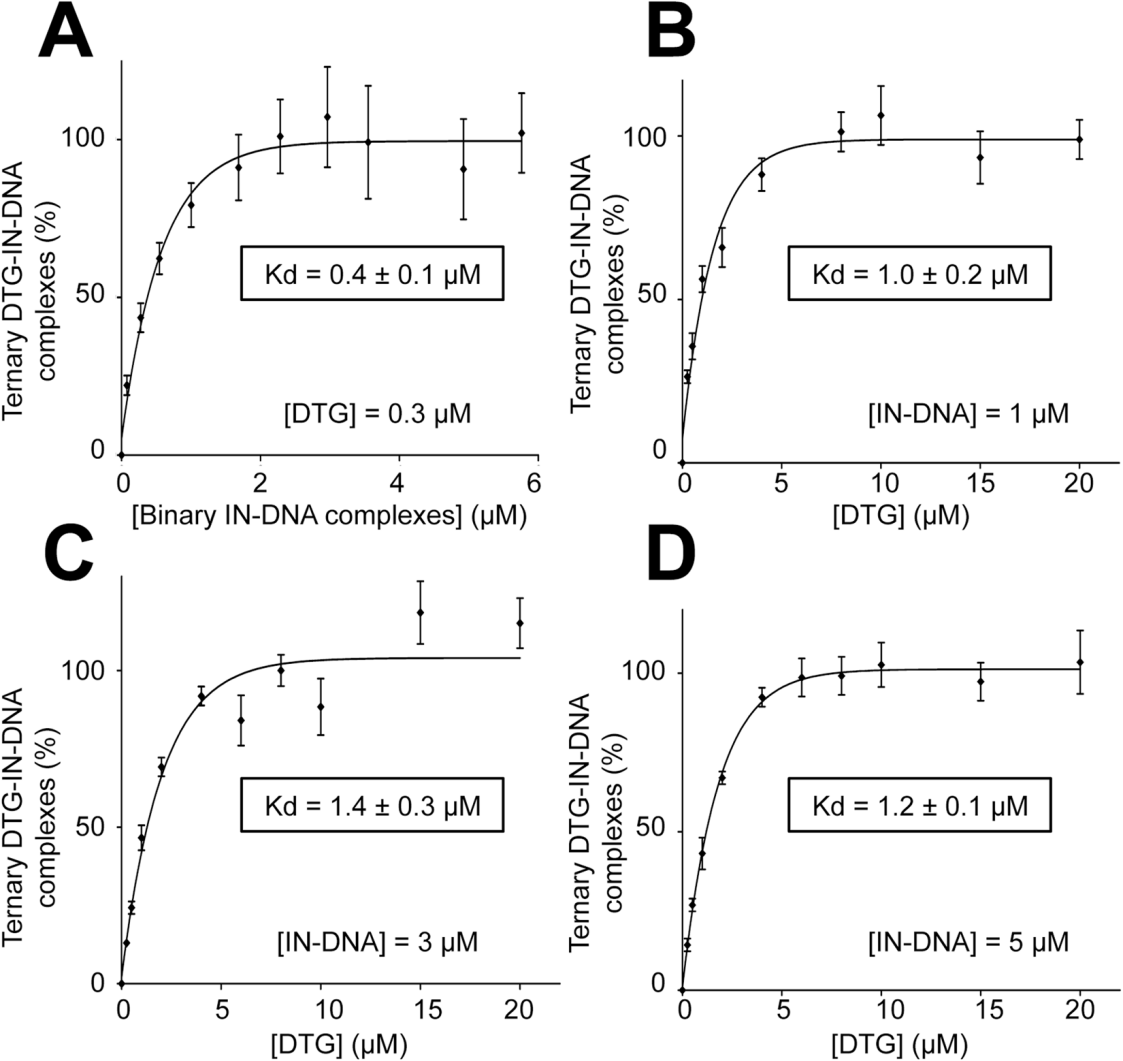
Measurement of the affinity of DTG for the binary INPFV-DNA complex. The formation of the ternary DTG-IN-DNA complex was measured in the presence of 1 mM Mg^2+^ by varying either the concentration of the binary IN-DNA complex (at a constant concentration of DTG: 0.3 μM) (**A**) or the DTG concentration (at a constant concentration of the binary complex: 1 μM (**B**), 3 μM (**C**) or 5 μM (**D**)). The percentage of ternary complexes (or % of bound DTG) were derived from fluorescence enhancement experiments as explained in the legend of Fig. 2C-D. λ_ex_ = 345 nm; λ_em_ = 375–385 nm; Δλ_ex_ = Δλ_em_ = 5 nm; PMT = 980 V (**A**), 750 V (**B**), 730 V (**C**), 710 V (**D**). The percentages of binary complexes were derived from fluorescence anisotropy experiments as explained in the legend of Fig. 2E. All graphs show mean ± SD values from three independent experiments.

To evaluate the K_d_ characterizing the interaction between DTG and the binary IN-viral DNA complex, we first calculated the number of IN-DNA complexes for each condition of stoichiometric concentrations of IN + PFV^CAAT^ by measuring the steady-state fluorescence anisotropy of Fl-PFV^CAAT^, the fluorescently labeled counterpart PFV^CAAT^ (Fig. 2E). The linear relationship between the formations of ternary DTG-IN-DNA and binary IN-DNA complexes (slope≈1; Fig. 2F) confirms that no DTG binding to IN alone or DNA alone occurs under our experimental conditions. This demonstrates that DTG binding to IN strictly requires the pre-formation of the IN-DNA complex in agreement with previous studies on INSTI compounds^37^. The quantification of DTG bound to the binary complex, based on fluorescence enhancement, was determined either by varying the concentration of binary complexes (for a given DTG concentration) or by varying DTG concentration (for a given concentration of binary complexes). Results were consistent and led to K_d_ values in the sub-to-low micromolar range (0.4–1.4 μM) for the ternary DTG-IN-DNA complex (Fig. 3).

Although the measurement of DTG fluorescence enhancement should account for binding events of DTG to Mg^2+^-IN-DNA complexes only, we checked that binding properties of DTG as measured by its intrinsic fluorescence, were not strongly influenced by the Mg^2+^ concentration of the sample. This is an important issue since 10 mM Mg^2+^ is optimal for IN activity whereas 1 mM Mg^2+^ corresponds to a sub-optimal condition (Supplementary Fig. S3). Similar experiments were then conducted using 10 mM Mg^2+^. As expected (see above), no significant DTG fluorescence emission enhancement was observed upon addition of IN-PVF^CAAT^ complexes, in contrast to results obtained with 1 mM Mg^2+^, although the blue-shift of the maximum emission wavelength was consistently observed (Supplementary Fig. S4A) accounting for a modest but significant increase in the DTG fluorescence intensity in the 375–385 nm region with 10 mM Mg^2+^ (see also Fig. 2B). Altogether, our results indicate that the proper Mg^2+^ complexation effect accounts for DTG emission intensity enhancement whereas the concomitant binding to the IN active site accounts for a classical emission wavelength blue-shift for polarity reasons^38^. This net increase, although modest compared to the 1 mM Mg^2+^ condition (resulting in titration curves of smaller amplitude; Supplementary Fig. S4A, inset), allowed an estimation of the K_d_ value for the ternary complex (≈0.7 μM) (Supplementary Fig. S4B–C) which is consistent with the value found in the presence of 1 mM Mg^2+^. However, if the fluorescence intensity appears to be suitable for measuring the formation of ternary complexes in the presence of 1 mM Mg^2+^, it appears suboptimal using 10 mM due to the lower amplitude of the enhancement process.

**Figure 4:**
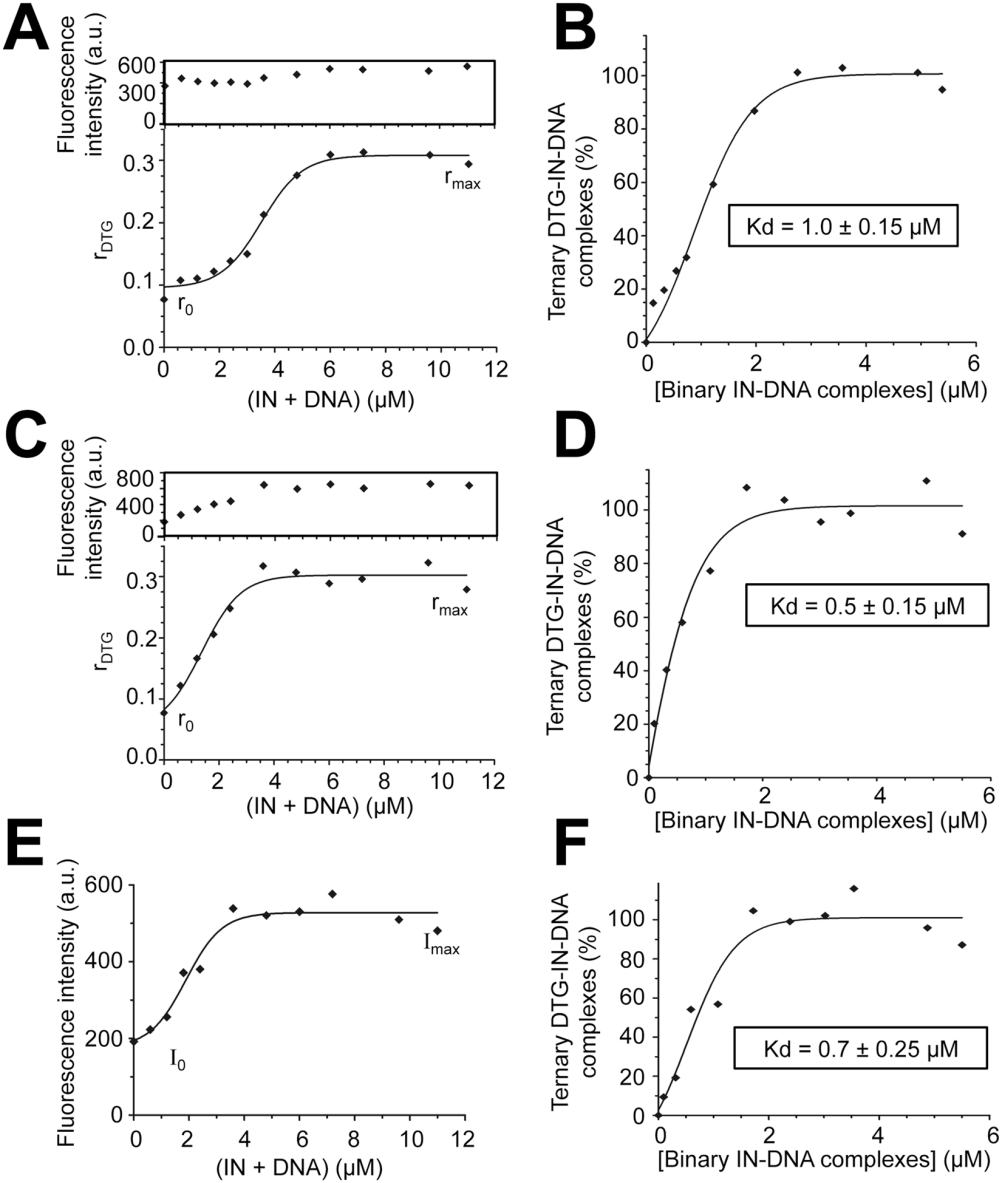
Measurement of DTG binding to the binary INPFV-DNA complex by steady-state fluorescence anisotropy. (**A** and **C**) Fluorescence anisotropy of DTG (0.6 μM) upon addition of increasing stoichiometric concentrations of (IN + DNA) in the presence of 10 mM (**A**) or 1 mM Mg^2+^ (**C**). λ_ex_ = 345 nm and PMT = 950 V (Δλ_ex_ = Δλ_em_ = 10 nm). Inset: concomitant change of the DTG fluorescence intensity. (**B** and **D**) Formation of the ternary DTG-IN-DNA complex as a function of the binary complex concentration in the presence of 10 (**B**) or 1 mM Mg^2+^ (**D**). The percentages of ternary complexes (or % of bound DTG) were derived from the plots shown in panels A & C, respectively: % = (r − r_0_)/[(r_max_ − r)(I_max_/I_0_) + (r − r_0_)] × 100 where r_0_ and r_max_ correspond to anisotropy values of free and bound DTG, respectively; I_0_ and I_max_ correspond to intensities of free and bound DTG, respectively (to account for the change in DTG intensity; see insets in panels A and C). The binary complex concentration was measured as explained in Fig. 2E. (E) Fluorescence intensity of a 0.6μM-DTG solution (integrated in the 375–385 nm spectral region) as a function of (IN + DNA). λ_ex_ = 345 nm and PMT = 980 V (Δλ_ex_ = Δλ_em_ = 5 nm). The DTG emission enhancement was used for the calculation of the percentage of ternary complexes (as explained in the legend of Fig. 2C–D). This percentage was then plotted against the concentration of the binary complex (**F**). The titration curve led to a K_d_ value of 0.7 ± 0.25 μM which is consistent with both (i) the value obtained with 0.3 μM DTG using the same approach (fluorescence enhancement): K_d_ = 0.4 ± 0.1 μM (Fig. 3A) and (ii) the value obtained with 0.6 μM DTG using the anisotropy-based approach: K_d_ = 0.5 ± 0.15 μM (panel D). All graphs show representative data of three (panels A and B) or two (panels C–F) independent experiments.

Alternatively, to overcome this difficulty, we measured the steady-state fluorescence anisotropy (r) of DTG itself in the presence of increasing concentrations of binary IN-PVF^CAAT^ complexes and 10 mM Mg^2+^. The rotational mobility of the drug should be constrained upon binding to its target. As shown in Fig. 4A, the r value continuously increased as a function of (IN + PFV^CAAT^), leading to a K_d_ value of 1.0 ± 0.15 μM characterizing the ternary complex (Fig. 4B). This value is consistent with the value determined either by fluorescence anisotropy with 1 mM Mg^2+^ (Fig. 4C–F) or by fluorescence intensity, regardless of Mg^2+^ concentration. Altogether, our results show that both DTG fluorescence intensity and anisotropy are suitable for the study of the interaction between DTG and the binary IN-DNA complex using 1 mM Mg^2+^ whereas mainly anisotropy is suitable using 10 mM Mg^2+^.

### Viral DNA sequence determinants for the binding of DTG to the binary IN-DNA complex

DTG binding to the binary IN-DNA complex with the 21-bp U5 LTR end (represented by the PVF^CAAT^ sequence mentioned above: DNA#1 in Table 1) was compared to other complexes obtained with 21-bp DNAs containing varying lengths of the cognate PFV sequence from the reactive strand 3′-end (for example: 16-bp in the case of DNA#2, 4-bp in the case of DNA#8; Table 1); DNA#9 corresponds to a non-specific (random) sequence. DTG binding to the different binary complexes was then measured using the fluorescence intensity procedure or the r parameter (columns 3 & 4; Table 1). DTG displayed good and similar binding activities to IN in complex with DNA#2→6 (>76% of the binding activity obtained with DNA#1) while a significantly lower binding activity was observed with DNA#7→9, suggesting that the 10 terminal base-pairs from the reactive strand 3′-end were sufficient to ensure DTG binding to the binary complex. Among these 10 positions, at least pos. 10 and the CA sequence (pos 3–4 preceding the 3′P cleavage site) are strictly required. Indeed, DTG binding to the binary complex was low using DNA#10 (CAAT-3′→AGGT-3′) and DNA#11 (CAAT-3′→AGAT-3′). By contrast, DTG binding was higher with DNA#12 (CAAT-3′→CAGT-3′) (72–77% of the binding activity obtained with DNA#1). This indicates that the nature of nucleotides at pos. 1/2 on both DNA strands is not crucial for DTG binding. Consistent with this observation, the pre-processed DNA substrate (DNA#13) led to efficient DTG binding which is expected since INSTIs that do not prevent 3′P, strongly inhibit the ST reaction^5^. To note, structural studies suggest an even higher stability or selectivity of the INSTI in the active site occupied by the processed DNA^39^. Such a situation is not observed in the present study since DTG binding in the DNA#13 context (processed LTR end) remains slightly lower compared to the DNA#1 context (blunt LTR end), in agreement with the lower ST inhibition efficiency of INSTI observed when using the pre-processed compared to the blunt substrate^40^. Moreover, INSTI such as RAL was shown to tightly bind to both processed and blunt LTR ends^41^. Altogether, results by us and others suggest that the active site may accommodate INSTI and blunt LTR end (*i.e*. LTR processing is not strictly required for INSTI binding) in a manner that not or minimally affects 3′P^42^. The apparent discrepancy with structure-based considerations could originate from the molecular nature of the complex considered, with the synaptic complex competent for the full-site integration reaction^39^ characterized by a higher selectivity toward the processed LTR end than the complex competent for the half-site ST reaction (present study and^40^).

**Table 1:** Binding of DTG to PFV IN complexed with various DNA sequences.

Name of the double-stranded DNA	Sequence of the reactive strand^a^ Pos: 21 20 19 18 17 16 15 14 13 12 11 10 9 8 7 6 5 4 3 2 1	DTG-binding (%)^b^	
		Fluorescence intensity procedure^c^	Fluorescence anisotropy procedure^d^
DNA#1	5′-**TAT ACA AAA TTC CAT GA** **C A** **AT**−3′	100	100
DNA#2	5′-GGA AT**A AAA TTC CAT GA** **C A** **AT**−3′	83 ± 5	n.d.^e^
DNA#3	5′-GGA ATC T**AA TTC CAT GA** **C A** **AT**−3′	89 ± 6	96 ± 3
DNA#4	5′-GGA ATC TAG **TTC CAT GA** **C A** **AT**−3′	84 ± 4	n.d.
DNA#5	5′-GGA ATC TAG C**TC CAT GA** **C A** **AT**−3′	101 ± 5	n.d.
DNA#6	5′-GGA ATC TAG CG**C CAT GA** **C A** **AT**−3′	76 ± 5	n.d.
DNA#7	5′-GGA ATC TAG CGG **CAT GA** **C A** **AT**−3′	20 ± 3	42 ± 6
DNA#8	5′-GGA ATC TAG CGG CGC AT**C A** **AT**−3′	7 ± 2	8 ± 2
DNA#9	5′-GGA ATC TAG CGG CGC ATA GGT-3′	9 ± 2	15 ± 3
DNA#10	5′-**TAT ACA AAA TTC CAT GA**A GGT-3′	23 ± 3	n.d
DNA#11	5′-**TAT ACA AAA TTC CAT GA**A G**AT**−3′	24 ± 2	29 ± 3
DNA#12	5′-**TAT ACA AAA TTC CAT GA** **C A**GT-3′	72 ± 6	77 ± 4
DNA#13^f^	5′-**TAT ACA AAA TTC CAT GA** **C A**−3′	66 ± 4	79 ± 5

^a^PFV nucleotides are in bold. The underlined sequence corresponds to the conserved retroviral dinucleotide sequence (CA) preceding the 3′-P cleavage site. ^b^Normalized by the cognate sequence (DNA#1). All experiments were performed with stoichiometric concentrations of IN and DNA (1.8 μM each). ^c^The procedure is indicated in Fig. 2C–D legend. [DTG] = 0.3 μM; [Mg^2+^] = 1 mM. ^d^The procedure is indicated in Fig. 4A–B legend. [DTG] = 0.6 μM; [Mg^2+^] = 10 mM. ^e^Non determined. ^f^19-mer oligonucleotide for mimicking the processed DNA product (when hybridized with the complementary sequence of DNA#1). All percentages are mean values ± SD from three independent experiments.

The results in Table 1 cannot discriminate between a direct sequence effect on DTG-binding from an indirect effect via a possible defect in the IN-DNA interaction. The binding of IN to various DNA sequences (DNA#1 #8 #9 and #13) was similar, as measured by fluorescence anisotropy using fluorescein-labeled DNAs (Supplementary Fig. S5A). However, we cannot rule out from the anisotropy assay, which quantifies the overall amount of IN-DNA complexes in the sample without any information regarding the positioning of IN onto DNA, that some sequence-dependent subtle changes in the specific interactions between IN and DNA may occur and thus indirectly perturb DTG binding. The X-ray structure of the IN^PFV^-DNA complex highlights specific contacts (about 9) between amino acids and the bases of the two strands (*i.e*. reactive and non-transferred DNA strands), up to the 10^th^ position from the reactive strand 3′-end^26^. In particular, there is a specific contact involving a guanine of the non-transferred strand at pos. 10 and the N69 residue. A modification of this base could perturb the positioning of IN onto DNA and, then indirectly prevent DTG binding (compare DNA#6 and #7; Table 1). Indeed, the significantly reduced ST activity using substrate DNA#7 (compared with the slight decrease observed with DNA#6; Supplementary Fig. S5B), combined to the absence of ST inhibition by DTG using IN-DNA#7 complexes while IN-DNA#1 and IN-DNA#6 complexes remained DTG-sensitive (Supplementary Fig. S5C), highlight a correlation between the capability of the binary IN-DNA complex to bind DTG and its catalytic property. This suggests that DTG binding requires a precise positioning of IN onto DNA ( = catalytically active complex). To note, based on X-ray structures of ternary RAL/or DTG-IN-DNA complexes^26,29^ in which the INSTI has direct contacts with several bases at pos. 3 and 4 (A3 and C4 on the reactive strand and G4 on the non-transferred strand) with an even closer contact between G4 and the DTG halogenated phenyl group^29^, the influence of these two positions could be more related to specific drug-DNA contacts.

### Monitoring the DTG-binding process to various IN mutants

**Figure 5:**
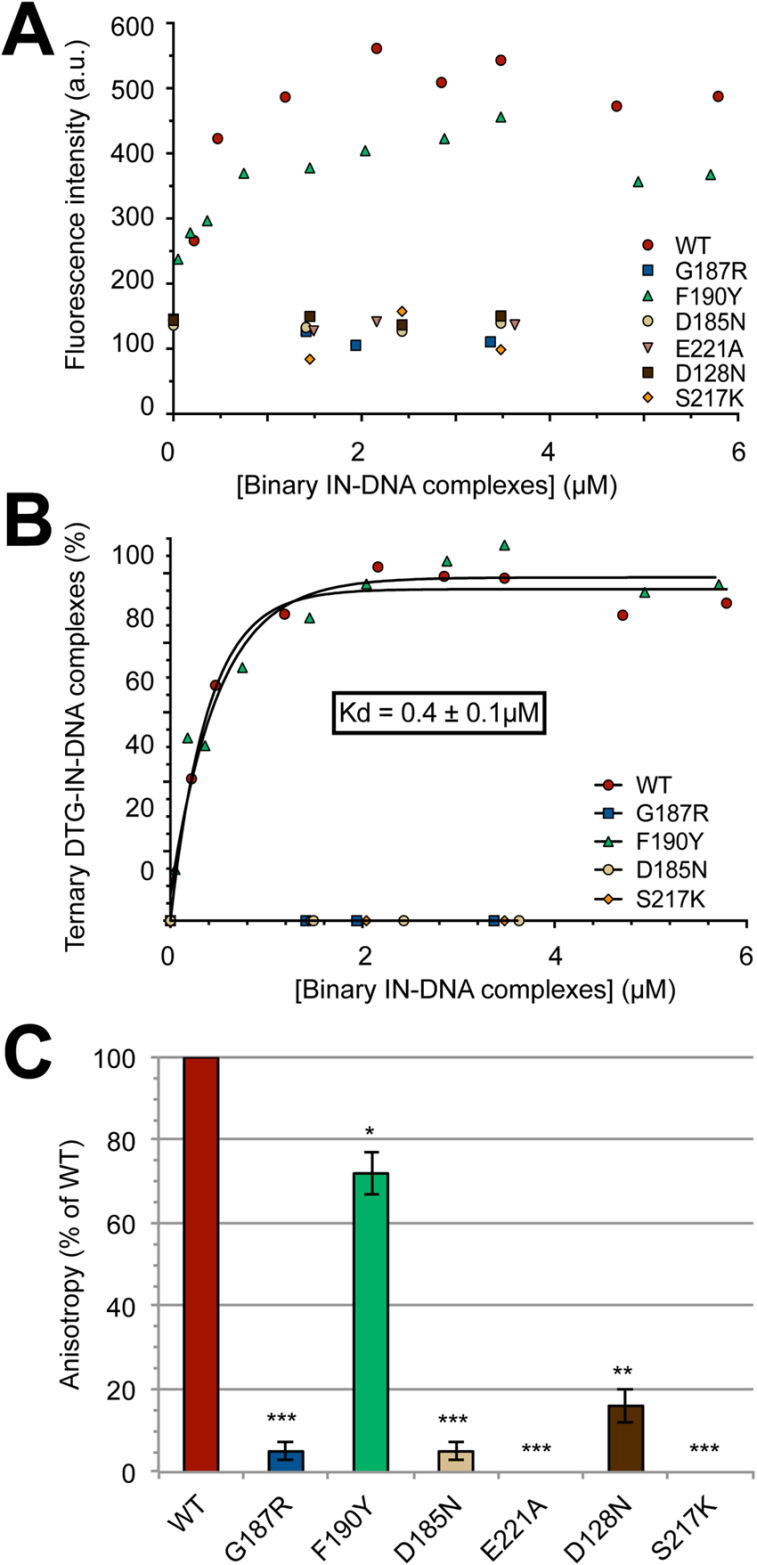
Measurement of DTG binding to various IN mutants complexed to the cognate viral DNA sequence. (**A**) DTG binding to the wt or mutant complexes was measured by monitoring DTG fluorescence enhancement (integrated in the 375–385 nm spectral region). λ_ex_ = 345 nm and PMT = 980 V (Δλ_ex_ = Δλ_em_ = 5 nm). The fluorescence intensity was monitored as a function of (IN + DNA) and plotted as a function of the binary IN-DNA complex concentration (measured as explained in Fig. 2E legend). [DTG] = 0.3 μM and [Mg^2+^] = 1 mM. (**B**) The percentage of ternary complexes was calculated using DTG fluorescence enhancement. For each protein, the DTG fluorescence was normalized by the maximal fluorescence intensity value, reached for an excess of binary IN-DNA complexes and corresponding to the plateau value in panel A. The results show that DTG displays similar affinities for wt and F190Y INs (K_d_≈0.4 μM), in contrast to that observed for G187R and S217K resistant mutants or the catalytic D185N mutant (the DNA-binding properties of mutants are shown in Supplementary Fig. S6). (**C**) Measurement of ternary DTG-IN-DNA complex formation for the wild-type IN^PFV^ and various mutants by monitoring DTG fluorescence anisotropy in the presence of 10 mM Mg^2+^. The steady-state fluorescence anisotropy of DTG (0.6 μM) was measured in the presence of stoichiometric concentrations of (IN + DNA): [IN] = [DNA] = 2.4 μM (see Fig. 4 legend for details about the instrumentation setting). The r value for each mutant was normalized by the value obtained for the wt: % anisotropy = [r_(DTG-mutant-DNA)_ − r_(free DTG)_]/[r_(DTG-wt-DNA)_ − r_(free DTG)_] × 100 where r_(DTG-mutant-DNA)_ and r_(DTG-wt-DNA)_ correspond to maximum fluorescence anisotropy values obtained for the mutant and the wt, respectively. This value is equal to ≈0.32 for the wt (=r_max_ in Fig. 4). r_(free DTG)_ corresponds to the fluorescence anisotropy of free Mg^2+^-DTG in solution (≈0.077 = r_o_ in Fig. 4). The graphs in panels A-B show representative data of three independent experiments. The bar graph in panel C shows mean ± SD values from three independent experiments, *p < 0.05, **p < 0.01, ***p < 0.001.

**Figure 6:**
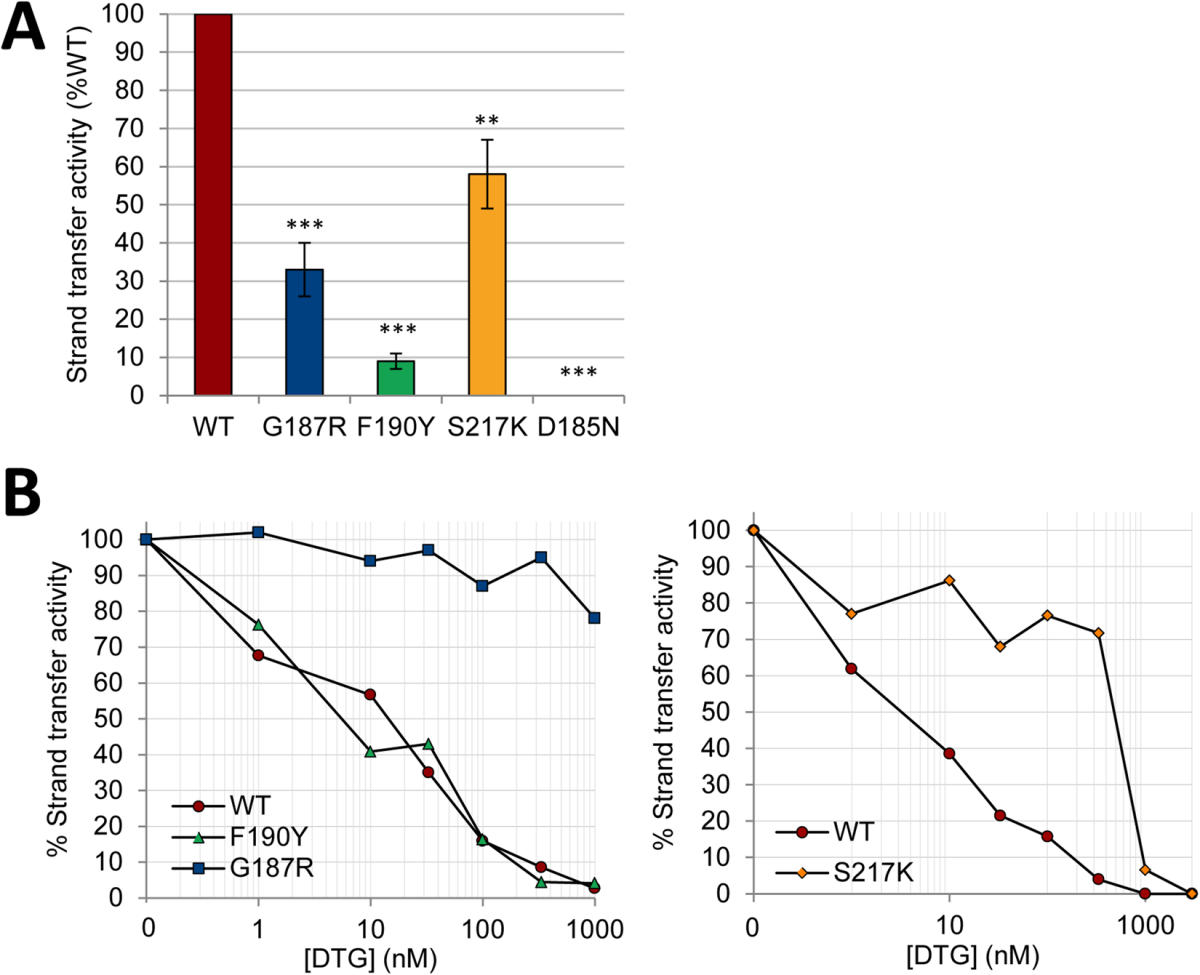
Strand transfer (ST) activities and measurements of resistance to DTG of G187R, F190Y and S217K INPFV mutants. (**A**) Relative ST activities of G187R, F190Y, S217K and D185N mutants (wt = 100%) in the absence of DTG. The bar graph shows mean ± SD values from three independent experiments, **p < 0.01, ***p < 0.001. (**B**) ST activities of wt, G187R, F190Y and S217K proteins as a function of DTG concentration (expressed for each protein as percentages of the value obtained for the condition without DTG). The graphs show representative data of three independent experiments. The ST activity of the wt IN^PFV^ or mutants was measured at 37 °C in the presence of 300 nM IN, 12.5 nM DNA#13 substrate (Table 1) and increasing concentrations of DTG. ST products were quantified as explained in the Methods section. The effect of DTG on the DNA-binding properties of IN is shown in Supplementary Fig. S7.

Recently, we have characterized two new single mutations involving G118 and F121 residues in IN^HIV^ that confer resistance against RAL and DTG, albeit to different extents (G118R being more resistant than F121Y)^24^. DTG binding to analogous G187R and F190Y IN^PFV^ was then investigated using fluorescence-based DTG-binding assays and compared to results obtained with the S217K IN^PFV^ mutant (equivalent to the RAL/DTG resistant G140S/Q148K IN^HIV^ double mutant^24^. No binding of DTG to G187R or S217K was observed whereas a slight but significant decrease of the plateau value was observed with F190Y (Fig. 5A). These results indicate dramatic effects of G→R and S→K mutations at pos. 187 and 217, respectively, on the ternary DTG-IN-DNA complex formation whereas we cannot exclude in the latter case an influence of the F→Y mutation on DTG emission in terms of polarity change, compatible with the close proximity of DTG and the phenyl ring of residue 190^29^. Consistent with this hypothesis, normalized DTG-binding curves were similar for the wt IN and F190Y indicating that the F→Y mutation did not directly and strongly influence DTG binding, in contrast to that observed with G187R or S217K (Fig. 5B). To note, the DTG-binding impairment, as observed for the G187R or S217K mutant, was not related to a net decrease of the total amount of IN-DNA complexes (Supplementary Fig. S6). We found that proteins susceptibility to DTG in the ST activity assay closely paralleled DTG-binding properties directly probed by the fluorescence-based DTG-binding assay (Fig. 6). S217K and G187R displayed highly resistant profiles (IC_50_ = 500 nM and >1 μM, respectively) while the wt IN^PFV^ and the F190Y mutant were similarly inhibited with IC_50_ values in the 5–15 nM range.

The DTG-binding defects of G187R and S217K mutants are apparently comparable to those observed for the three catalytic triad mutants (D128N, D185N and E221A corresponding to HIV-1 D64N, D116N and E152A, respectively) (Fig. 5A–B). Taking into account that Mg^2+^ concentration is suboptimal in the fluorescence intensity-based assay (1 mM), the measurement of DTG-binding was repeated in the presence of 10 mM Mg^2+^ by monitoring DTG anisotropy as described above: the DTG-binding defects of G187R and S217K were consistently observed in this condition (Fig. 5C). The mechanism behind the loss of DTG-binding for the catalytic mutants most likely relies on the loss of Mg^2+^ chelation since the three catalytic residues are involved in the chelation of at least one of the two Mg^2+^ in the context of the IN-DNA complex^26^. To note, Mg^2+^ is dispensable for the overall DNA-binding step of IN^32,43–45^ and thus, the loss of Mg^2+^ chelation is not contradictory with results showing that catalytic mutants bind equally well to DNA and in a similar manner than wt IN (Supplementary Fig. S6). However, G187R cannot be considered as a catalytic mutant since it sustains a significant ST activity although weak (≈10% of the wt activity; Fig. 6). In the case of S217K, the ST activity was significantly higher (≈60% of the wt activity; Fig. 6).

Interestingly, X-ray structures of S217Q/H mutants show a loss of metal binding suggesting that the presence of a bulky side chain could be responsible for a reduction in cofactor binding affinity due a displacement of the D185 residue^28^. However, authors exclude that this could be a direct explanation for the decreased INSTI susceptibility of S217H because S217Q and wt IN^PFV^ displays similar INST susceptibilities; they show that S217H requires a larger backbone conformational change in the active site, representing a higher energy cost to accommodate the 2^nd^-generation inhibitor MK2048 compared with S217Q^28^. Taking into account that (i) DTG and MK2048 share similar binding modes in the IN active site^29^ and (ii) the decrease in DTG susceptibility is significantly higher for S217K than S217H (≈50-fold and 2–3-fold, respectively) (Fig. 6)^29^, we can reasonably assume a common mechanism of resistance for S217K/H mutants with a steric hindrance effect which is even more accentuated in the case of the S→K substitution, explaining the dramatic DTG-binding defect of the S217K mutant. Based on the strongly reduced activity of G187R compared to the wt IN or S217K, we cannot totally exclude a problem of Mg^2+^ chelation for G187R that could be indirectly responsible for the DTG-binding defect. Not mutually exclusive with such an indirect effect of the G→R mutation, a direct effect (*i.e*. loss of an IN-drug contact) is also likely to be involved, consistent with X-ray structures of ternary drug-IN-DNA complexes showing that DTG as well as MK2048 extend toward the G187 residue via the tricyclic metal-chelating cores^29^.

In conclusion, the Mg^2+^-dependent DTG fluorescence emission allowed the development of two fluorescence-based assays to directly probe DTG binding to the binary IN-DNA complex. The first one is based on the fluorescence enhancement/blue shifted emission of the drug upon Mg^2+^ complexation and requires suboptimal (relative to IN activity) Mg^2+^ concentrations (*e.g*. 1 mM). The second one is based on the measurement of DTG fluorescence anisotropy that significantly increases upon its binding to the binary complex. In contrast to the first approach, the anisotropy-based approach is not limited by Mg^2+^ concentration and is compatible with optimal Mg^2+^ concentrations for IN activity (*e.g*. 10 mM). However, due to the use of polarizers that inherently diminishes detection sensitivity, the second approach requires higher DTG concentrations than the first one (typically 0.6 *vs* 0.3 μM). Nevertheless, the two approaches are consistent with each other, although not really surprising since only DTG in the context of Mg^2+^-IN-DNA leads to measurable fluorescence emission and then, only DTG-binding events occurring in this context are monitored, regardless of the Mg^2+^ concentration (optimal or suboptimal).

IC_50_ values characterizing RAL, EVG and DTG are in the low-nanomolar range^18,24,46^, below the concentrations used here in the fluorescence-based assay (0.3–0.6 μM) and below the K_d_ value (sub/low-micromolar) characterizing the binding of DTG to the binary IN-DNA complex. Such a K_d_ value is comparable to values obtained for competitive inhibitors (INBI: IN-binding inhibitors) such as styrylquinolines^47,48^, which are much less efficient inhibitors (IC_50_ values in the low-micromolar range) than noncompetitive INSTIs. This raises the question of how similar K_d_ can lead to large differences in inhibition values? First, the targets are clearly distinct for INBIs and INSTIs: free IN and the binary complex, respectively. Taking into account that IN activity assays require a large excess of IN over DNA, the concentration of target is then much higher for INBIs than for INSTIs. Another (not mutually exclusive) explanation could be related to the larger population of complexes probed in fluorescence experiments (mixture of IN-DNA_donor_ and IN-DNA_donor_-DNA_acceptor_ complexes) compared to ST activity experiments (IN-DNA_donor_-DNA_acceptor_ complexes only) with an enhanced affinity of the drug in the presence of the acceptor (or target) DNA. Importantly, our results on mutations of the LTR end (from the DNA side) and resistance mutations (on the protein side) show that the modulations of DTG fluorescence properties remain suitable to predict DNA-binding and/or DTG-binding properties in relationship with both structural and functional studies. Therefore, although (i) DNA concentrations used in fluorescence experiments are larger than those used in standard 3′P/ST activity tests (sub/low-micromolar range and low-nanomolar range, respectively) and (ii) DTG concentrations are larger than IC_50_ values, the experimental conditions remain compatible with probing the binding of DTG to the binary complex because, most importantly, the corresponding K_d_ value and DTG concentration used are in the same range.

Nevertheless, a possible limitation could be that INSTIs do not behave similarly - in terms of mechanism of action - toward the IN-viral DNA complex in the 0.3–0.6 and 0.01–0.1 μM drug concentration ranges (corresponding to typical concentrations used in the fluorescence-based assay and IC_50_ values, respectively). The inhibition of IN-DNA interactions by INSTIs was then tested over a wide concentration range (up to 20 μM). Their incapability to prevent or disrupt IN-viral DNA complexes was consistently observed over the whole concentration range, regardless of the INSTI tested (Supplementary Fig. S7). Moreover, the intrinsic ability of INSTI to also inhibit 3′P activity has been recently described but this occurs only above micromolar drug concentrations^49,50^. All considerations mentioned above explain why the fluorescence-based DTG-binding assay remains robust and predictive of both DNA-binding properties and resistance mutations of IN and could be extended to the study of other resistance mutations against DTG or EVG, the anisotropy mode being more suitable in the latter case since EVG fluorescence emission is not strongly dependent on Mg^2+^ chelation.

## Methods

### Oligonucleotides for DTG- and DNA-binding assays, INSTI compounds

Unlabeled and fluorescein(Fl)-labeled DNAs were purchased from Eurogentec (Belgium) and further purified on polyacrylamide gel^25^. Sequences and names of DNAs are reported in Table 1. Dolutegravir, raltegravir and elvitegravir were generous gifts from Pr. Vincent Calvez (Hopital Pitié-Salpêtrière, Paris).

### Site-directed mutagenesis and purification of IN^PFV^ mutants

IN^PFV^ mutants (D128N, D185N, E221A, G187R, F190Y and S217K) were constructed using the QuikChange Lightning site-directed mutagenesis kit (Agilent) according to manufacturer’s instructions. The pET-IN-PFV plasmid containing the full-length IN gene was used for mutagenesis^25^. Nucleotide sequences of mutagenic primers are explicitly shown in Supplementary Table S1. The resulting plasmids were prepared after transformation of *E. coli* XL10-Gold ultracompetent cells (Agilent) (cells were grown in LB medium containing 100 μg/ml ampicillin at 37 °C) using the QIAprep Spin Miniprep kit (Quiagen) and were sequenced (Eurofins Genomics) across the entire IN-encoding region to ensure that no unintended nucleotide change was introduced during the mutagenesis procedure.

The full-length wt IN^PFV^, the three single mutants of the catalytic triad (D128N, D185N, E221A) and RAL/DTG-resistant mutants (G187R, F190Y, S217K) were expressed using *E. coli* BL21-CodonPlus (DE3)-RIPL cells (Agilent) (cells were grown in LB medium containing 100 μg/ml ampicillin at 37 °C) and purified under native conditions. The purification was based on a batch procedure by using Ni-NTA agarose beads (Qiagen). At an OD (600 nm) of 0.6–0.8, His-tagged PFV IN expression was induced in bacterial cultures by the addition of IPTG (1 mM) and cultured for additional 4 h at 37 °C. Cells were harvested by centrifugation and frozen at −20 °C. Cells from 1 liter culture were resuspended in 24 ml of ice-cold filtered purification buffer (20 mM Tris-HCl pH 8, 1 M NaCl, 4 mM β-mercaptoethanol) supplemented with 5 mM imidazole (Im) and EDTA-free Protease Inhibitor Cocktail (Roche)). Cells were lysed in a French press and centrifuged (30 min at 10,000 rpm). The supernatant was filtered (0.45μm pore size) and incubated overnight with Ni-NTA agarose beads (Qiagen). The beads were washed twice with 10 vol. of purification buffer + 5 mM Im, 10 vol. of purification buffer + 50 mM Im, and 10 vol. of purification buffer + 100 mM Im. IN was then eluted with purification buffer supplemented with 50 μM ZnSO_4_ and 1 M Im. Im was removed by overnight dialysis against purification buffer supplemented with 50 μM ZnSO_4_ and 10% ethylene glycol (v/v). Fractions were aliquoted and frozen at −80 °C.

### Characterizations of IN^PFV^ ST activity and susceptibility to DTG

DNA#13 mimicking the 3′-processed end (Table 1) was radiolabeled with T4 polynucleotide kinase (NEB) and [γ-^32^P]ATP (Amersham) and purified on a Sephadex G-10 column. The double-stranded DNA substrate was obtained by mixing equimolar amounts of ssDNA#13 with its 21-mer complementary strand in the presence of 100 mM NaCl. ST reactions were carried out at 37 °C with 200 or 300 nM IN in 20 mM HEPES pH 6.8, 1 mM DTT, 10 mM MgCl_2_ and 50 mM NaCl, in the presence of 12.5 nM DNA. After separation in a 16% acrylamide/urea denaturing gel, ST products were analyzed with a Typhoon TRIO variable mode imager (GE Healthcare) and quantified with ImageQuant TL software. ST activity was expressed as the % of the signal intensity of ST products divided by the signal intensity of the DNA substrate. DTG susceptibilities of wt IN and mutants were determined by measuring ST activity in the presence of increasing drug concentrations. IC_50_ values were determined using the Prism 5.0 software.

### Measurements of IN-drug or (IN-DNA)-drug interactions by steady-state fluorescence intensity and anisotropy

Fluorescence excitation and emission spectra of INSTIs were recorded on a Eclipse (Varian) spectrofluorimeter equipped with two polarizers for anisotropy measurements and a thermostated cell holder, using 80 µl solutions placed in micro cells (Hellma). Drug, IN and DNA concentrations, excitation/emission slit settings as well as the photomultiplier tube (PMT) voltage are specified in figure legends. The intensity value was corrected from the inner filter effect. The steady-state fluorescence anisotropy was calculated using: r = (I_VV_–G x I_VH_)/(I_VV_ + 2GxI_VH_) where I_VV_ and I_VH_ correspond to vertical and horizontal components, respectively, when the sample is excited with vertically polarized light. G represents the correction factor for the difference in the monochromator transmission between // and ⊥ polarized components: G = I_HV_/I_HH_. All experiments were performed at 25 °C in buffer A: 20 mM Tris pH 7.2, 50 mM NaCl, 1 mM DTT and 10% (v/v) DMSO.

### Measurement of the IN-DNA interaction by steady-state fluorescence anisotropy

IN^PFV^ binding to DNA was measured by fluorescence anisotropy on a Beacon Instrument (Panvera, Madison, WI, USA) as previously described^25,51^, using a double-stranded 21-mer Fl-labeled DNA mimicking the PFV U5 LTR end also referred to as DNA#1 in Table 1 (labeled at the 5′-end of the reactive strand). IN binding to Fl-PFV^CAAT^ (or Fl-DNA#1) was compared to the binding to other DNA sequences (Fl-DNA#8 #9 #13). DNA annealing was obtained by mixing equimolar amounts of complementary strands in buffer A, heating to 85 °C for 5 min and slow cooling to 25 °C. DNA-binding experiments were performed at 25 °C in buffer A.

### Statistical analysis

Statistical significance was determined using the paired t-test and two-way ANOVA (GraphPad Prism 5).

## Electronic supplementary material

Supplementary Information
