# Supplementary material for: Probing Resistance Mutations in Retroviral Integrases by Direct Measurement of Dolutegravir Fluorescence
Source: Sci Rep. 2017 Oct 25;7:14067. doi: 10.1038/s41598-017-14564-w (PMC5656594; doi:10.1038/s41598-017-14564-w)
Supplement: Supplementary file 1 — Supplementary Information [file 41598_2017_14564_MOESM1_ESM.pdf]

SUPPLEMENTARY INFORMATION

Probing Resistance Mutations in Retroviral Integrases by Direct Measurement of Dolutegravir Fluorescence

Eloïse Thierry[1], Samuel Lebourgeois[1], Françoise Simon[1], Olivier Delelis[1,*], and Eric Deprez[1,*]

[1]Laboratory of Biology and Applied Pharmacology (LBPA), CNRS UMR8113, IDA FR3242, ENS Paris-Saclay, Université Paris-Saclay, F-94235 Cachan, France

[*]Correspondence and requests for materials should be addressed to O.D. (email: delelis@lbpa.ens-cachan.fr) or E.D. (email: deprez@lbpa.ens-cachan.fr)

Table of contents:

- Figure S1……………………………………………………………page 2
- Figure S2……………………………………………………………page 3
- Figure S3……………………………………………………………page 4
- Figure S4……………………………………………………………page 5
- Figure S5……………………………………………………………page 6
- Figure S6…………………………………………………………… page 7
- Figure S7……………………………………………………………page 8
- Table S1……………………………………………………………page 9

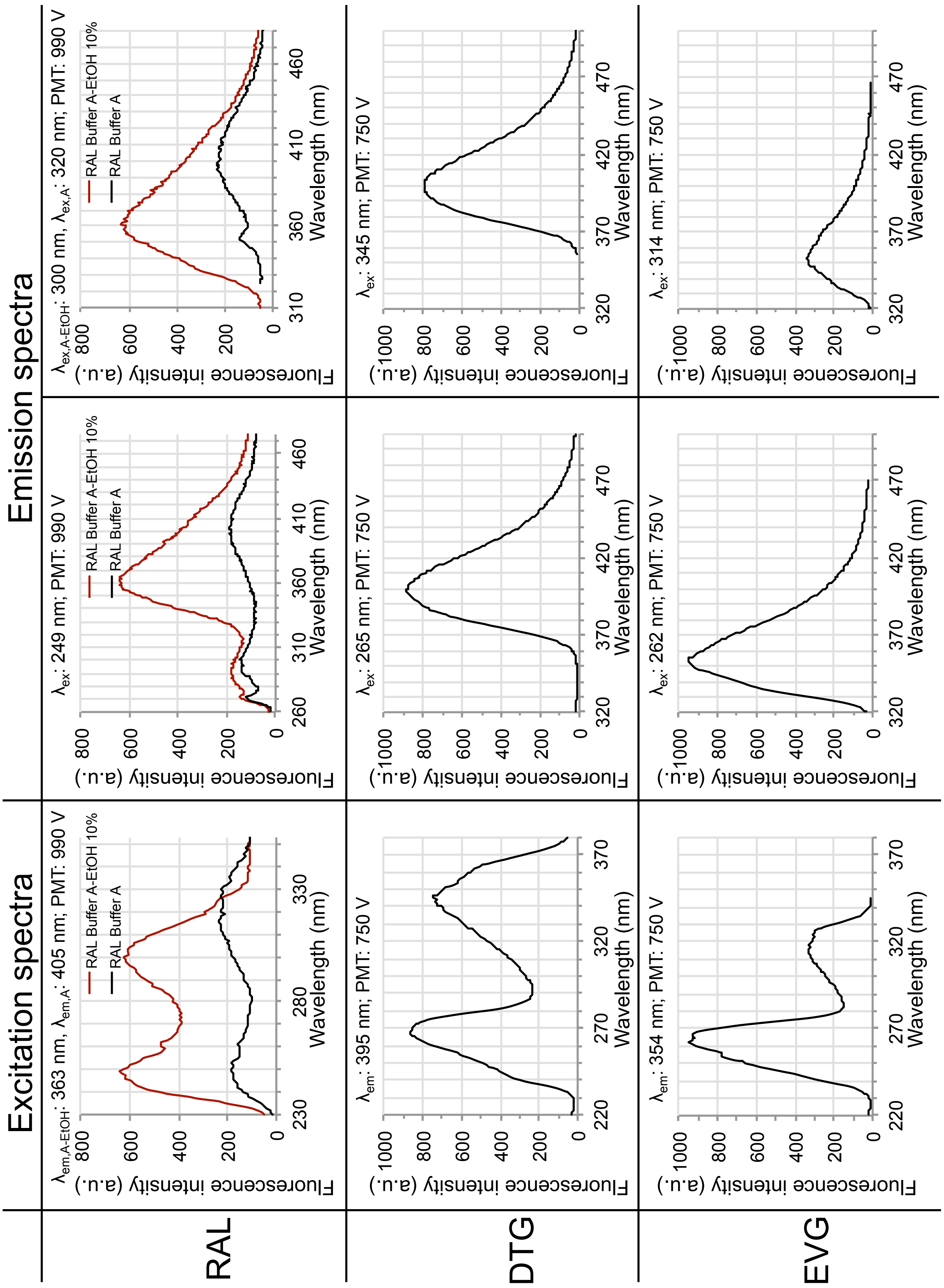

Figure S1. Excitation and emission spectra of INSTIs in solution. INSTIs (final concentration, 10 μM) were diluted into the buffer A supplemented with 10 mM Mg^{2+} except RAL which was also studied in buffer A-ethanol 10% (v/v) + 10 mM Mg^{2+} (in red). λ_{ex}, λ_{em} and PMT voltage are indicated in the figure legend. The excitation and emission slits were 5 nm for all spectra.

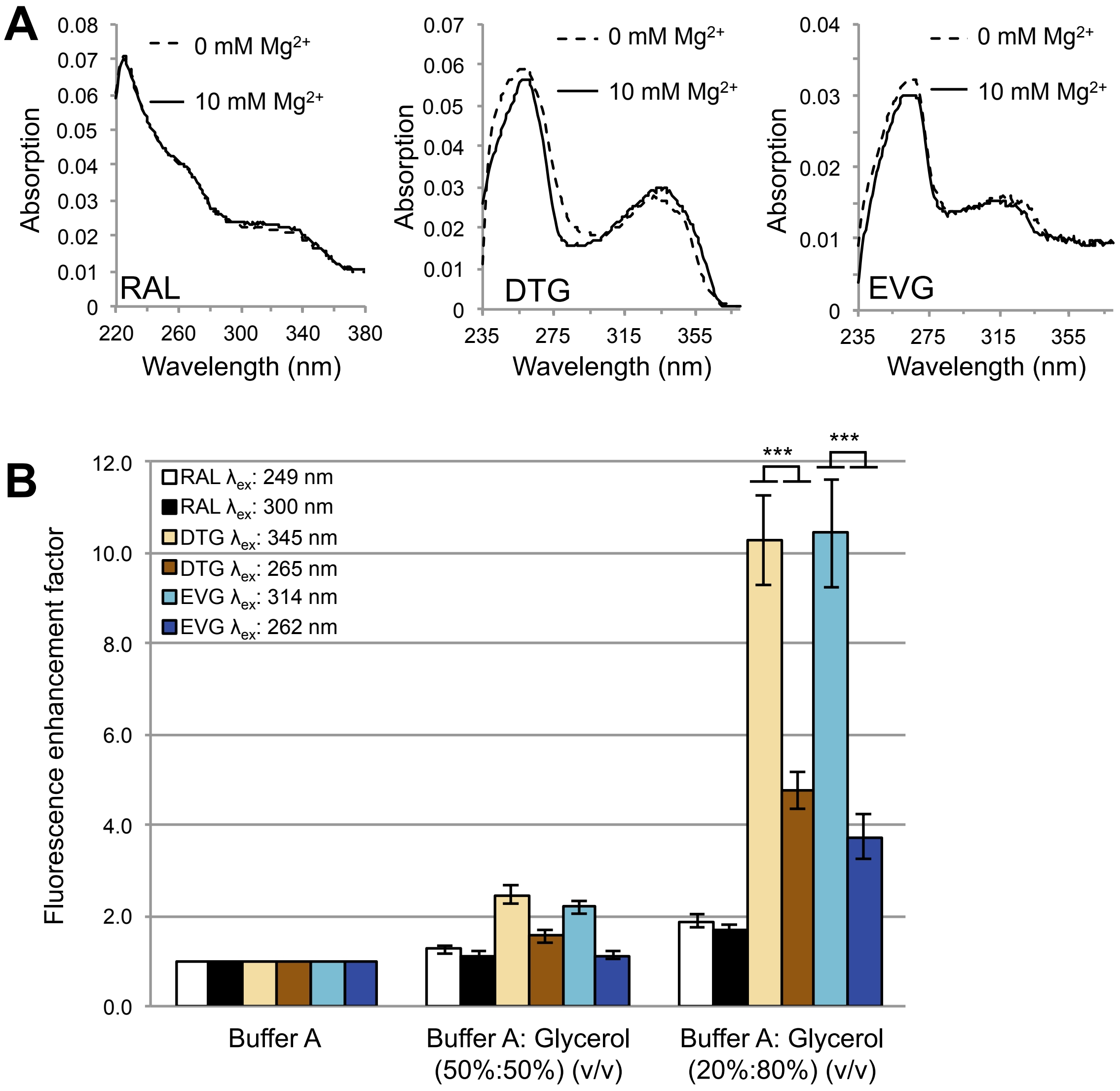

Figure S2. Effects of Mg^{2+} on absorption spectra and of solvent viscosity on fluorescence enhancement of INSTIs. (**A**) INSTIs (final concentration, 3 μM) were diluted into the buffer A with or without 10 mM Mg^{2+}. (**B**) Fluorescence enhancement factor of RAL ($\lambda_{max,em}$ = 363 nm), EVG ($\lambda_{max,em}$ = 354 nm) and DTG ($\lambda_{max,em}$ = 395 nm) as a function of the excitation wavelength and glycerol concentration. The excitation and emission slits were 5 nm (PMT = 750V for DTG and EVG; PMT = 990V for RAL). The fluorescence enhancement factor corresponds to the drug fluorescence emission intensity in the presence of glycerol normalized by the intensity in the absence of glycerol. The bar graph shows mean ± SD values from three independent experiments, ***$p<0.001$.

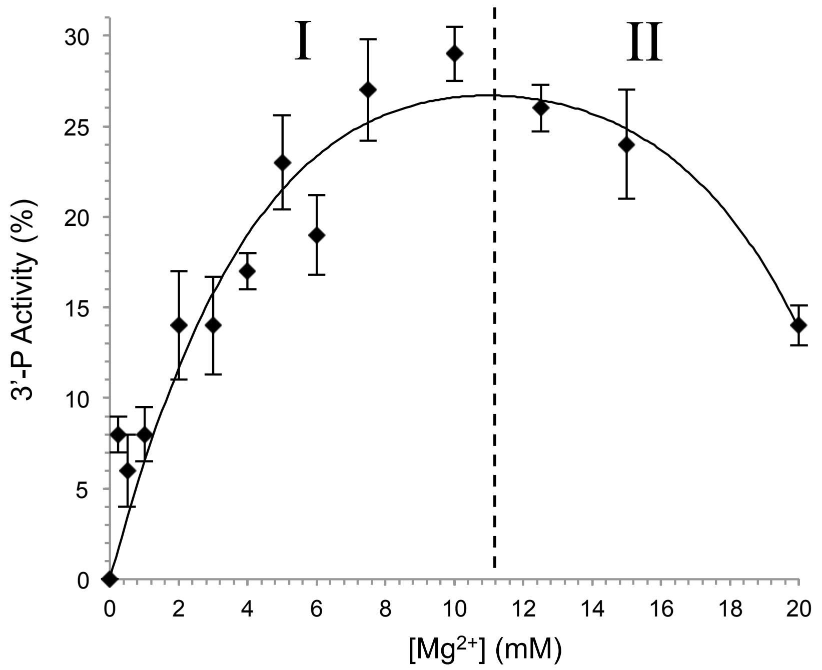

Figure S3. 3'-processing activity of IN^{PFV} as a function of Mg^{2+} concentration. The 3'P assay was carried out according to[25,32] using the 21-mer PFV^{CAAT} DNA substrate (also named DNA#1; Table 1) fluorescently labeled at the 3'-end of the reactive strand. The 3'P activity was measured in buffer A using 4 nM of DNA in the presence of 400 nM IN^{PFV} and increasing Mg^{2+} concentrations. The bell-shaped curve clearly indicates an increasing phase (I) which corresponds in first approximation to the titration curve for the binding of Mg^{2+} to IN, followed by a decreasing phase (II) which indicates a weakened IN/DNA interaction upon increasing ionic strength consistent with previous results[25]. The graph shows mean ± SD values from two independent experiments. The phase I (before the dashed line) was analysed by using a classical rectangular hyperbolic equation for the estimation of the K_d value characterizing the Mg^{2+}-IN interaction: 2.0 ± 0.4 mM.

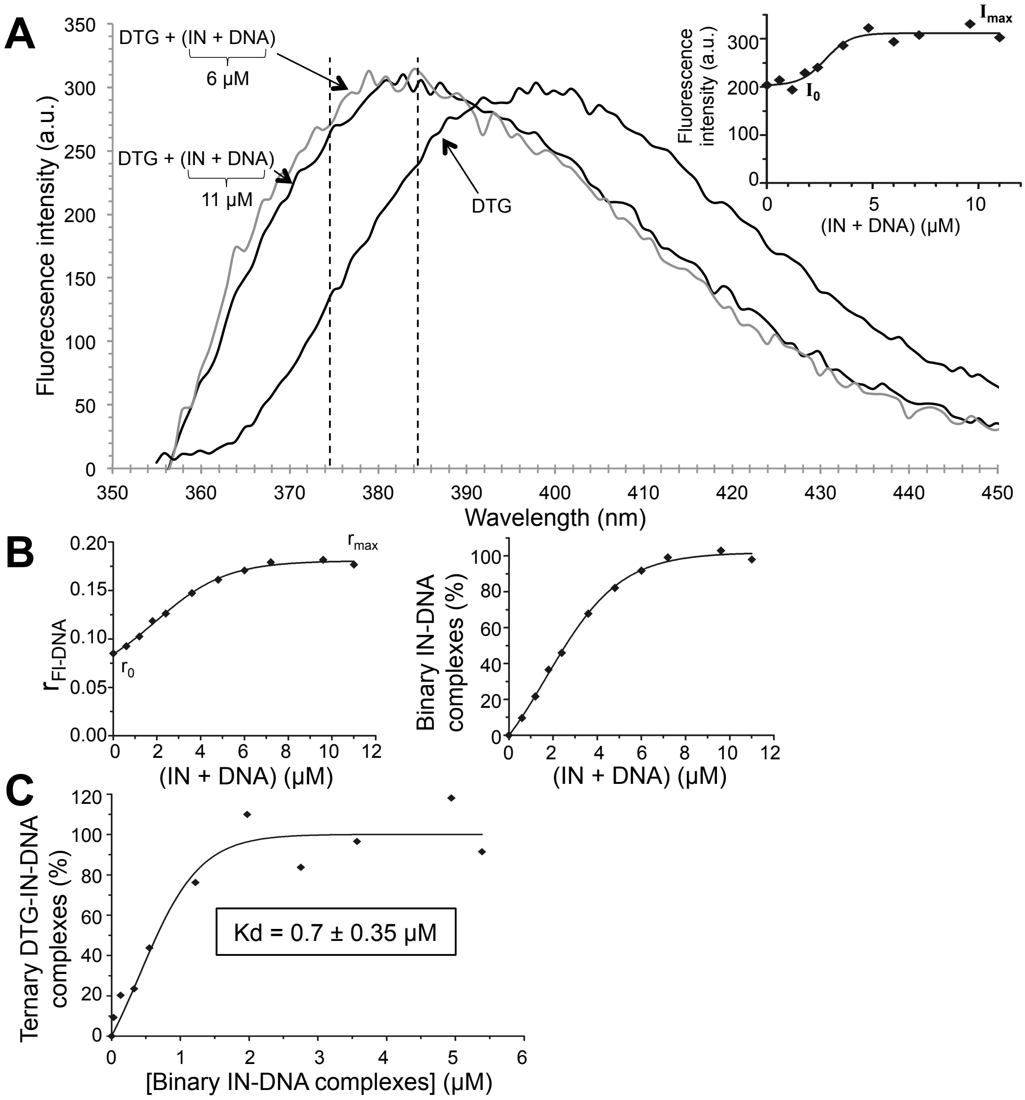

Figure S4. Fluorescence properties of DTG in the context of the binary IN^{PFV}-viral DNA complex, in the presence of 10mM Mg^{2+}. (**A**) Fluorescence emission spectra of DTG (0.3 μM) alone or in the presence of both IN and PFV^{CAAT} DNA ([IN] = [DNA] = 3 μM or [IN] = [DNA] =5.5 μM). All experiments were performed in the presence of 10 mM Mg^{2+}, using λ_{ex} = 345 nm ($\Delta\lambda_{ex} = \Delta\lambda_{em}$ = 5 nm) and PMT = 980V. Inset: fluorescence intensity of DTG (integrated in the 375-385 nm spectral region; delineated by the dashed lines) as a function of (IN+DNA). (**B**) Measurement of the binary IN-DNA complex formation by monitoring the steady-state fluorescence anisotropy (r) of DNA under similar experimental conditions of the DTG-binding assay (panel A). Left, varying stoichiometric concentrations of IN and DNA were mixed and the r parameter was monitored. A constant concentration of Fl-PFV^{CAAT} (0.3 μM) was maintained throughout the titration. Right, percentage of binary complexes as a function of (IN+DNA): $\%=(r-r_0)/(r_{max}-r_0)x100$ where r_0 and r_{max} correspond to anisotropy values of free DNA and IN-bound DNA, respectively. (**C**) Formation of ternary DTG-IN-DNA complexes as a function of the binary complex concentration. The percentage of ternary complexes (or % of bound DTG) was derived from the inset figure in panel A: $\%= (I-I_0)/(I_{max}-I_0)x100$ where I_0 and I_{max} correspond to fluorescence intensities of DTG alone and in the presence of an excess concentration of (IN+DNA) (= plateau value), respectively. The concentrations of binary complexes were deduced from fluorescence anisotropy experiments as shown in panel B. All graphs show representative data of two independent experiments.

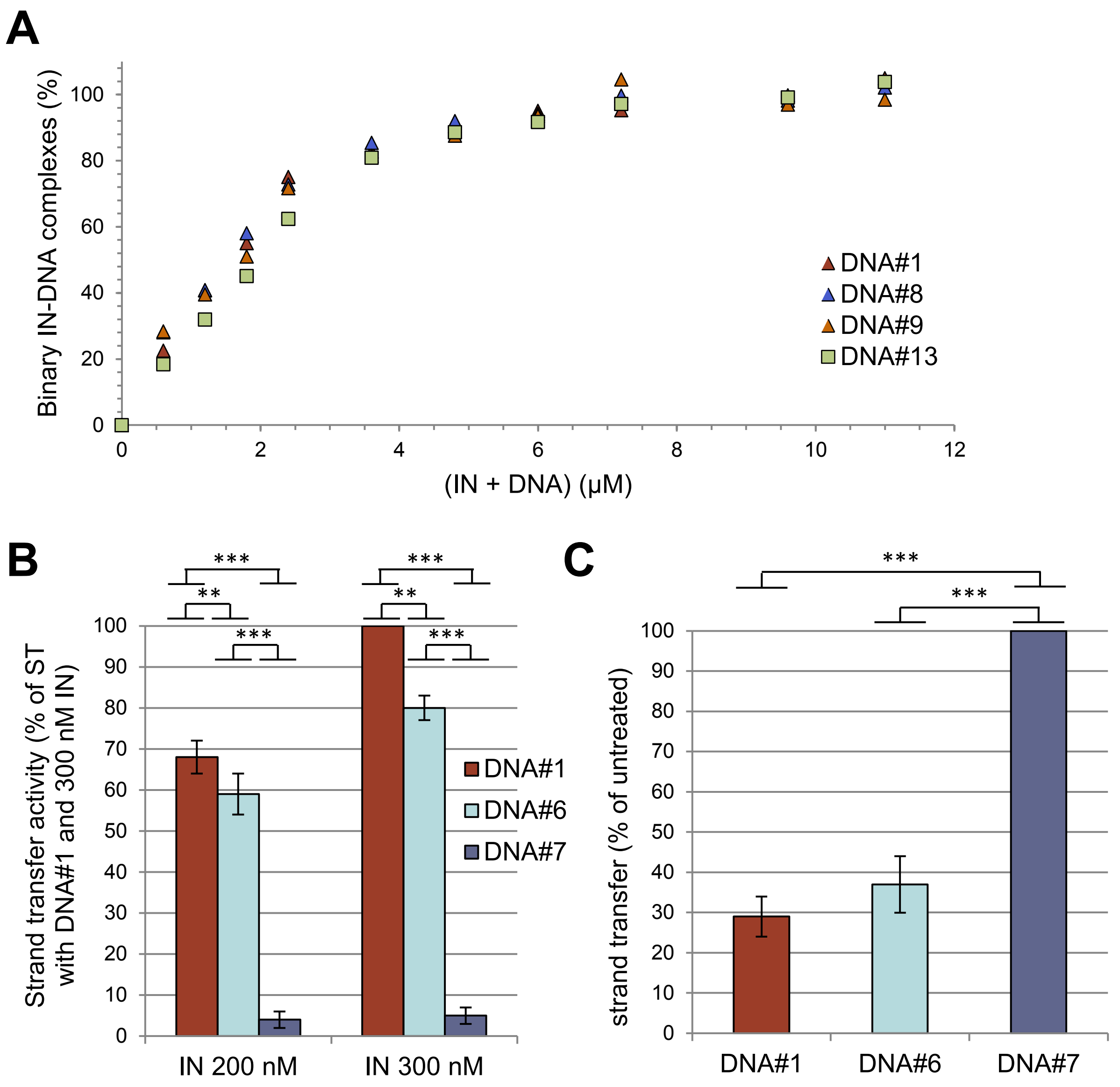

Figure S5. Comparison of the binary IN^{PFV}-DNA complex formation using various DNA sequences. (**A**) The binding of IN^{PFV} to various DNA sequences was studied by steady-state fluorescence anisotropy as indicated in Fig. 2E legend, using fluorescein-labeled counterparts of 21-mer DNA#1, #8, #9 and #13 (sequences are indicated in Table 1). Varying stoichiometric concentrations of IN and DNA were mixed in the presence of 1mM Mg^{2+} and the r parameter was monitored. A constant concentration of Fl-DNA (0.3 µM) was maintained throughout the titration. The percentage of binary complexes as a function of (IN+DNA) was calculated using: $\%=(r-r_0)/(r_{max}-r_0)$ x100 where r_0 and r_{max} correspond to anisotropy values of free DNA and IN-bound DNA, respectively. The graph shows representative data of two independent experiments. (**B**) ST activity of IN (200 or 300 nM) after 4h incubation at 37°C using radiolabeled DNA#1, #6 or #7. Results are expressed as percentage of the activity corresponding to: DNA#1 + 300 nM IN. (**C**) Inhibition of the ST activity by 100 nM DTG using DNA#1, DNA#6 or DNA#7. IN concentration was 300 nM. For each substrate, results are expressed as the percentage of activity obtained without DTG. The bar graphs in panels B-C show mean ± SD values from three independent experiments, $^{**}p<0.01$, $^{***}p<0.001$.

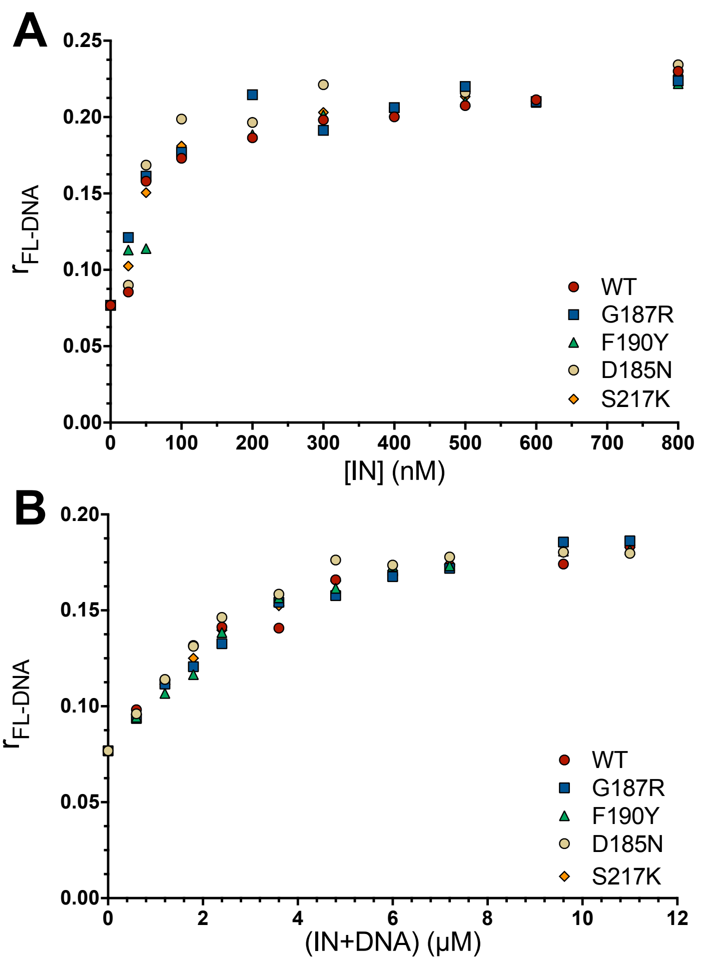

Figure S6. Comparison of DNA-binding properties between wild-type IN^{PFV} and various mutants. The formation of IN-DNA complexes was measured by steady-state fluorescence anisotropy using Fl-PFV^{CAAT} (also named Fl-DNA#1) in the presence of 1 mM Mg^{2+}. The measurement of the DNA-binding process was performed either under standard titration conditions (Panel **A**: varying concentrations of IN were added to a constant concentration of Fl-DNA#1 (= 4 nM) according to[25,51] or under similar experimental conditions than those described for the DTG-binding assay (Panel **B**: varying stoichiometric concentrations of IN and DNA were mixed with a constant concentration of Fl-DNA#1 (= 0.3 μM) maintained throughout the titration). The graphs show representative data of two independent experiments. Regarding the catalytic mutants, E221A & D128N (not shown) display similar DNA-binding profiles than D185N (explicitly shown).

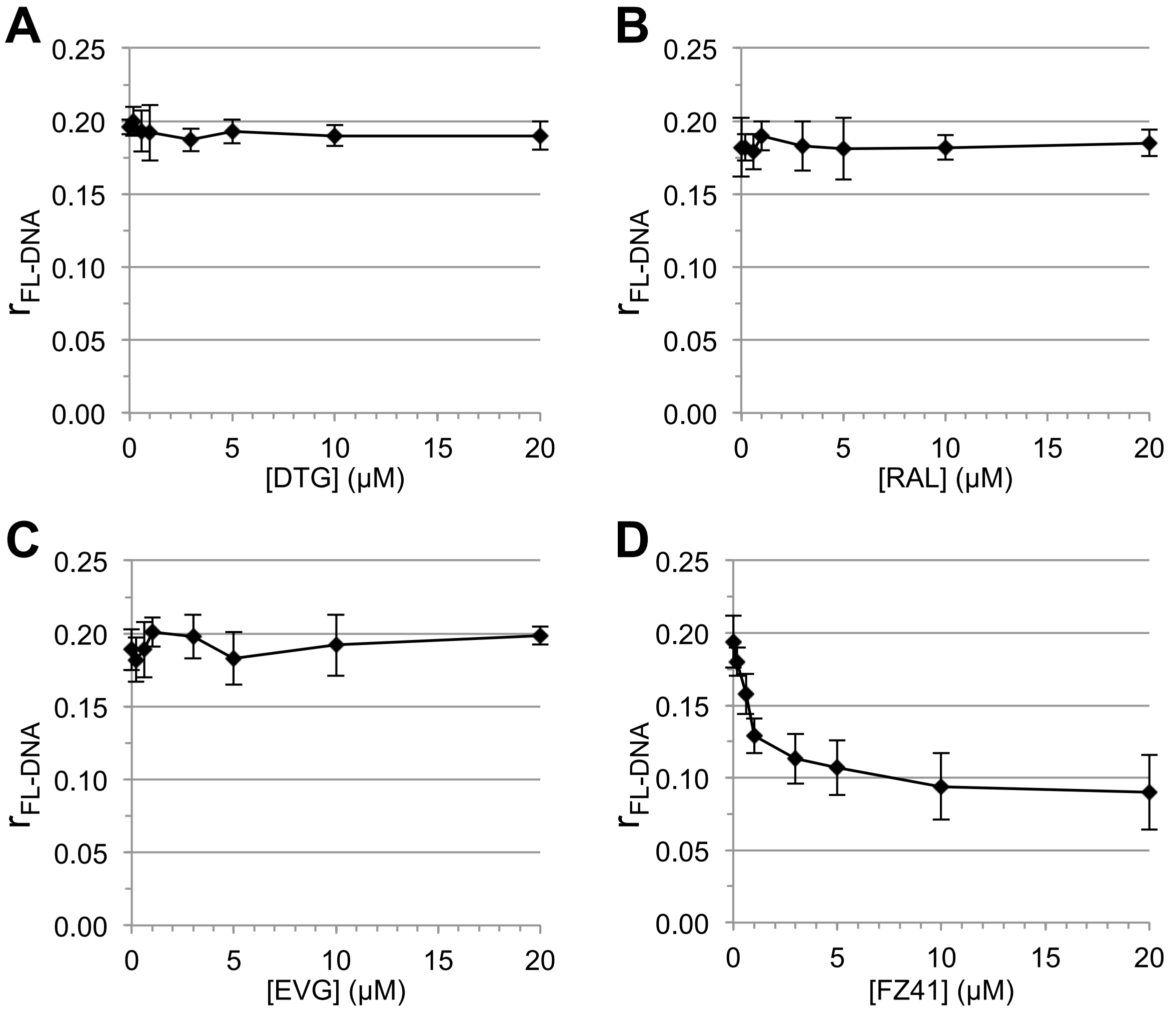

Figure S7. Differential effect of INSTI and INBI compounds on the IN^{PFV}-viral DNA interaction. The formation of IN-DNA complexes was measured by steady-state fluorescence anisotropy using 400 nM IN^{PFV} and 4 nM Fl-PFV^{CAAT} (also named Fl-DNA#1) in the presence of 10 mM Mg^{2+} and increasing drug concentrations: DTG (**A**), RAL (**B**), EVG (**C**) or FZ41 (**D**). In each plot, the starting r value (≈ 0.20) corresponds to the typical value found at saturating concentrations of IN (see Fig. S6). This value remained constant by increasing INSTI concentration (A-C), indicating that INSTI compounds did not prevent IN-DNA interactions over the whole concentration range (up to 20 µM). The INBI compound FZ41, a member of the styrylquinoline family of inhibitors, was used as a control and displays a competitive inhibition pattern with an IC_{50} of ≈ 0.8 µM, according to[32,51]. The graphs show mean ± SD values from two independent experiments.

Table S1. Nucleotide sequences of mutagenic primers used for site-directed mutagenesis of PFV IN.

Name of the mutant	Oligonucleotide
D128N PFV	5'-CCT TTT GAT AAA TTC TTT ATT AAC TAT ATT GGA CCT TTG CCA CC-3'
	5'-GGT GGC AAA GGT CCA ATA TAG TTA ATA AAG AAT TTA TCA AAA GG-3'
E221A PFV	5'-CCC CCA AAG TGG TAG TAA GGT GGC AAG GAA AAA TAG TGA-3'
	5'-TCA CTA TTT TTC CTT GCC ACC TTA CTA CCA CTT TGG GGG-3'
D185N PFV	5'-CCA AAG GTG ATT CAC TCT AAT CAA GGT GCA GCA TTC ACT-3'
	5'-AGT GAA TGC ACC TTG ATT AGA GTG AAT CAC CTT TGG-3'
F190Y PFV	5’- AGC AAA GGT TGA AGA AGT GTA TGC TGC ACC TTG ATC AGA-3’
	5’- TCT GAT CAA GGT GCA GCA TAC ACT TCT TCA ACC TTT GCT-3’
G187R PFV	5’- TGA AGA AGT GAA TGC TGC ACG TTG ATC AGA GTG AAT CAC-3’
	5’- GTG ATT CAC TCT GAT CAA CGT GCA GCA TTC ACT TCT TCA-3’
S217K PFV	5’- TTT CCT TTC CAC CTT ACC TTT ACT TTG GGG GTG ATA AGG-3’
	5'-CCT TAT CAC CCC CAA AGT AAA GGT AAG GTG GAA AGG AAA-3'
